# The antibacterial potency and antibacterial mechanism of a commercially available surface‐anchoring quaternary ammonium salt (SAQAS)‐based biocide in vitro

**DOI:** 10.1111/jam.15729

**Published:** 2022-07-31

**Authors:** Shilpa Saseendran Nair, Vikash Anand, Karnika De Silva, Siouxsie Wiles, Simon Swift

**Affiliations:** ^1^ Department of Molecular Medicine and Pathology Waipapa Taumata Rau University of Auckland Auckland New Zealand; ^2^ NZ Product Accelerator, Faculty of Engineering Waipapa Taumata Rau University of Auckland Auckland New Zealand

**Keywords:** antibacterial mechanism, fomite transmission, membrane depolarization, membrane permeability, organosilicon quaternary ammonium compound, reactive oxygen species (ROS), surface anchoring biocide

## Abstract

**Aims:**

To determine the antimicrobial potency of a surface‐anchored quaternary ammonium salt (SAQAS)‐based biocide during in vitro wet and dry fomite assays and to determine the mechanism of killing bacteria on the surface.

**Methods and Results:**

Wet and dry fomite assays were established in vitro for a commercially available biocide (SAQAS‐A) applied to glass and low‐density polyethylene (LDPE) surfaces. Both wet and dry fomite tests showed the active killing of Gram‐positive and Gram‐negative bacteria but not endospores. Assays measuring membrane permeability (ATP and DNA release), bacterial membrane potential and bacterial ROS production were correlated with the time‐to‐kill profiles to show SAQAS‐A activity in suspension and applied to a surface.

**Conclusions:**

SAQAS‐A is an effective biocide against model strains of vegetative bacteria. The killing mechanism for SAQAS‐A observed minimal membrane depolarization, a surge in ROS production and assessment of membrane permeability supported the puncture of cells in both suspension and surface attachment, leading to cell death.

**Significance and Impact of the study:**

SAQAS represents effective surface biocides against single challenges with bacteria through a mechanical killing ability that supports real‐world application if their durability can be demonstrated to maintain residual activity.

## INTRODUCTION

Hospital‐acquired infections (HAI) are an immediate threat to patient health in developed and developing countries (Allegranzi et al., 2011; Arefian et al., 2016; Zhou et al., 2019). In New Zealand, HAIs are a significant cause of morbidity in hospitalized patients, with cumulative incidence reported at 6.33% in 2003 (Graves et al., 2003) and 10.7% in 2015 (Read & Bhally, 2015), rates that are mirrored in hospitals for all developed countries (Read & Bhally, 2015). The hospital environment, including various surfaces (faucet handles, switches, mobile phones (Shahaby et al., 2012), computer keyboards (Bures et al., 2000), door handles, bed rails, and benchtops) has been implicated as one factor responsible for the transmission of hospital‐acquired pathogens to patients (Ahmed et al., 2019; Lemmen et al., 2004; Russotto et al., 2015; Weber et al., 2010). One approach to accomplish a much‐needed reduction in HAI may be by preventing the survival of pathogens in the hospital environment, especially as the long‐term survival of HAI‐pathogens on surfaces (Hota, 2004; Kramer et al., 2006; Neely & Maley, 2000; Otter et al., 2013) can lead to outbreaks.

The globally accepted microbial burden on fomites in hospitals is <1 colony‐forming unit (CFU) per cm^2^ for antibiotic‐resistant and spore‐forming organisms, while the aerobic colony count should be <5 CFU per cm^2^ (Dancer, 2004; Malik et al., 2003; Schmidt et al., 2012). Chemical disinfection of surfaces is in routine use but has a number of limitations; it is rarely 100% effective, there is resistance to specific compounds (Andersen, 2016; Chapman, 2003; Langsrud et al., 2003), cleaning protocols or recommended disinfectant concentrations may be inefficient (Russotto et al., 2015) and disinfected surfaces are open to recolonization (Alfa, 2019; Russotto et al., 2015). Self‐disinfecting surfaces have been proposed to overcome some of the limitations of disinfection processes to reduce surface transmission (Kaur & Liu, 2016). Contact killing at surfaces through the application of antimicrobial polymers is receiving attention, and surface‐anchored quaternary ammonium compounds (Gong et al., 2012; Li et al., 2016) are a well‐documented class of biocides in this category.

The antimicrobial activity of 3‐(trimethoxysilyl)‐propyldimethyloctadecyl ammonium chloride, (CAS 27668–52‐6; C_26_H_58_ClNO_3_Si), an organosilicon quaternary ammonium compound (Si‐QAC), was first investigated by Isquith et al. (1972). The Si‐QAC silane group condenses with free hydroxyl groups on the surface (Sambhy et al., 2008), and intermolecular siloxane (Si‐O‐Si) linkages (Legido‐Quigley et al., 2003) form to stably anchor the biocide at the surface (Mathew, Cooney, Malmstrom, et al., 2018; Mathew, Cooney, Zujovic, et al., 2018), giving rise to the name surface anchoring quaternary ammonium salt (SAQAS). The antimicrobial mechanism is assigned to the positively charged quaternary amine (N+ atom) that attracts the negatively charged microbes onto the needle‐like C18 structure of the hydrophobic chain, which penetrates the cell envelope of micro‐organisms. This intimate interaction is proposed to lead to the denaturation of proteins in the bacterial cell membrane (McDonnell & Russell, 1999; Oblak et al., 2019) and disturbs the electrical balance and osmotic pressure of the bacterial membrane leading to cell lysis (Daood & Yiu, 2018; Xu et al., 2012). Other studies suggest that killing occurs through an ion‐exchange mechanism in the bacterial cell membrane (Crismaru et al., 2011; Mathew, Cooney, Zujovic, et al., 2018).

In recent years, several commercially available products have become available that include a Si‐QAC as the active agent in an aqueous spray‐on formulation. The products often claim surface protection for an extended period of time, commonly in the order of 1 month. In this study, we test the hypothesis that Si‐QAC‐treated surfaces will effectively kill bacteria through a mechanism that punctures the cell envelope (small holes) leading to larger holes and cell lysis. We first test the activity of a representative, readily available Si‐QAC product (Zoono Z‐71 Microbe Shield Surface Sanitiser) in wet and dry fomite tests against antibiotic‐sensitive and antibiotic‐resistant strains of a Gram‐positive bacterium (*Staphylococcus aureus*), a Gram‐negative bacterium (*Escherichia coli*), and bacterial endospores (*Bacillus cereus*). The wet fomite test is based on a standard testing method (JIS Z 2801, 2010) and inoculates the challenge bacteria in saline covered by an impervious and inert polymer film to give an even spread and good contact with the surface while avoiding desiccation stress. The dry fomite test inoculates the challenge bacteria in 1 μl aliquots that quickly dry onto the surface; killing may be a combination of the biocidal surface and desiccation.

Secondly, we investigate the mechanism of biocide killing in suspension and anchored to a surface using a panel of simple tests. The timing and extent of bacterial death (Ghosh & Haldar, 2020; Hartmann et al., 2010) determined by culture‐based methods are correlated with effects on membrane permeability measured by quantification of the release of ATP through small pores formed in the cell membrane; and of DNA, through larger holes formed in the bacterial cell envelope (Ioannou et al., 2007; Zapotoczna et al., 2017). We further assess the dissipation of the bacterial membrane potential, possibly due to pore formation (Zapotoczna et al., 2017), and measure the potential development of oxidative stress by quantifying intracellular reactive oxygen species (ROS) production (Dwyer et al., 2014).

## MATERIALS AND METHODS

### Bacterial strains

The bacterial strains used in this study are *Bacillus cereus* ATCC 10702 (New Zealand Reference Culture Collection: Medical Section [NZRM], the Institute of Environmental Science and Research Limited, New Zealand); *Escherichia coli* ATCC 25922 (American Type Culture Collection [ATCC] via Cryosite, Australia); Extended‐spectrum beta‐lactamase (ESBL) *E. coli* CTX‐M‐14 (Clinical isolate, Auckland Hospital); *Staphylococcus aureus* ATCC 6538 (ATCC via Cryosite, Australia) and Methicillin‐resistant *S. aureus* (MRSA) FPR3757 (Clinical isolate, Auckland Hospital). Strains were stored as frozen stocks at −80°C and recovered on Difco Tryptic Soy Agar (TSA, Fort Richard, Auckland) with incubation at 37°C overnight. Broth cultures were prepared by inoculating 10 ml of Difco Tryptic Soy Broth (TSB, Fort Richard) with a loopful of culture from a TSA plate and incubation at 37°C overnight with shaking at 200 rev min^−1^ *. Bacillus cereus* endospore preparation followed a standard method (ASTM Standard, 2012).

### Preparation of antimicrobial surfaces

A commercially available, spray‐on Si‐QAC biocide formulation Zoono Z‐71 Microbe Shield Surface Sanitiser (Elitepac NZ Ltd.) was used, containing the siloxane anchoring 3‐(trimethoxysilyl)‐propyidimethyloctadecyl ammonium chloride, termed here as SAQAS‐A (Surface Anchoring Quaternary Ammonium Salt‐A). Glass surfaces were presterilized by autoclaving. Low‐density polyethylene (LDPE) surfaces were presterilized by sequential 60 s immersions in 100%, 70% (v/v in water) and 100% ethanol, allowed to dry in a biosafety cabinet and treated with a commercial preparation of SAQAS‐A biocide using a spray method. One spray application was allowed to dry on the surface, and then two consecutive sprays were applied and allowed to dry, with roughly 500 μl of biocide formulation required to cover a surface area of 5 cm^2^. Untreated surfaces were used as a control.

### Testing of antimicrobial surfaces

For wet fomite testing, a methodology based on the Japanese Industrial Standard method 2801:2010 (JIS Z 2801, 2010) was used to quantify the killing ability of nonporous biocidal surfaces. Briefly, surfaces were challenged with approximately 10^6^ CFU of bacteria or endospores in saline. LDPE surfaces (carrier dimensions: 5 cm × 5 cm) were inoculated with 10^6^ CFU in 100 μl and covered with a sterile LDPE (4 cm × 4 cm) square. Glass surfaces (7.5 cm × 2.5 cm) were inoculated with 10^6^ CFU in 10 μl and covered with a 2.5 cm × 2.5 cm piece of sterile LDPE.

For dry fomite testing, an established methodology was used (Warnes & Keevil, 2011), where an inoculum of approximately 10^6^ CFU in 1 μl was used for both glass and LDPE carriers (2.5 cm × 2.5 cm) and allowed to dry (5 min) onto the test surface in a Class II biosafety cabinet.

Wet and dry fomite challenges were carried out at room temperature (20 ± 2°C) to be consistent with real‐world applications. After incubation, viable bacteria were recovered after contact with the test surface for one of the five‐time points (10 min, 30 min, 1, 2 and 20 h) by vortex mixing for 10–15 s in Letheen Broth (Merck Millipore Corporation) in a 50 ml V‐bottomed tube and enumerated as CFUs in dilutions following overnight incubation at 37°C on TSA. Three technical repeats were performed for each of three independent biological replicates for each combination of the test (wet or dry fomite), surface and bacterial strain.

### MIC and MBC Testing

Biocide dilutions were made in BBL Mueller Hinton II Broth (Cation‐Adjusted) (MHB; Fort Richard) in a 96‐well microplate and inoculated with a bacterial suspension diluted from an overnight culture in MHB to approximately 10^6^ CFU per ml. Microplates were incubated overnight at 37°C under constant shaking of 200 rev min^−1^, with the minimum inhibitory concentration (MIC) recorded by the eye as the lowest biocide concentration in the wells showing no growth. To determine the minimum bactericidal concentration (MBC), 10 μl from the MIC‐test wells with no observable growth was plated to Mueller‐Hinton Agar (MHA) and incubated at 37°C overnight. MBC values were determined as the lowest concentration returning no bacterial growth.

### ATP release assay

Bacterial suspensions at approximately 10^6^ CFU per ml were challenged with lethal (2 × MBC) and sublethal (1/4 × MIC) biocide concentrations in 10 ml at 37°C under constant shaking at 200 rev min^−1^, with 1 ml aliquots taken before biocide addition (time 0) and 10 min, 1 and 3 h after biocide addition. Cell‐free supernatants were collected from each aliquot after centrifugation at 19,330 × *g* for 2 min with ATP release quantified in 10 μl from each tube using a commercial ATP determination kit (A22066, Thermo Fisher Scientific) according to the manufacturer's instructions. Positive (polymyxin‐B, Sigma‐Aldrich; MIC_*E. coli*_ 3.1 μg/ml, MBC_*E. coli*_ 12.5 μg/ml, MIC_*S. aureus*_ 50 μg/ml, MBC_*S. aureus*_ 200 μg/ml) and negative (bacteria only and biocide only) controls were used for the test, and three biological replicates were conducted with three technical triplicates.

For bacteria in contact with a surface, SAQAS‐A treated glass coverslips (13 mm diameter) were placed inside wells of a 24‐well plate (Falcon). A bacterial suspension of approximately 10^6^ CFU (in 1 μl saline) was added to test, or untreated control, surfaces; 1 μl aliquots of the suspension were used as time 0 samples. The 24‐well plates were incubated at room temperature for 10 min, 1 and 3 h, with 1 ml of saline was added to each well to recover ATP released from the bacterial cells and quantified in 10 μl using the commercial ATP determination kit.

### Nucleic acid release assay

Bacterial suspensions at approximately 10^6^ CFU per ml were challenged with lethal (2 × MBC) and sublethal (1/4 × MIC) biocide concentrations in 10 ml at 37°C under constant shaking at 200 rev min^−1^, with 1 ml aliquots taken before biocide addition (time 0) and 10 min, 1 and 3 h after biocide addition. Cell‐free supernatants were collected from each aliquot after centrifugation at 19,330 × *g* for 2 min with 400 μl samples passed through 0.2 μm filters before measuring DNA concentrations using the Nanodrop 2000 Spectrophotometer (Thermo Fisher Scientific) platform at 260 nm. Triplicate readings were taken for aliquots from each sample on three separate test‐samples using saline as blank and polymyxin B as a positive control.

### Membrane potential assay

Bacterial suspensions at approximately 10^6^ CFU per ml were challenged with lethal (2 × MBC) and sublethal (1/4 × MIC) biocide concentrations in 10 ml at 37°C under constant shaking at 200 rev min^−1^, with 500 μl aliquots taken before biocide addition (time 0) and 10 min, 1 and 3 h after biocide addition and diluted with 500 μl of phosphate‐buffered saline (Thermo Fisher Scientific). The membrane potential was measured using a BacLight™ Membrane Potential Kit (B34950, Thermo Fisher Scientific) according to the manufacturer's instructions, after staining with DiOC_2_ and measuring the proportion of red and green cells by flow cytometry (BD LSR II Flow Cytometry, BD Biosciences). Bacteria treated with CCCP were used as a depolarized control according to the BacLight™ Membrane Potential Kit instructions. The test was conducted in three replicate test‐samples with measurements on technical triplicates.

### ROS assay

Bacterial suspensions at approximately 10^6^ CFU per ml were challenged with lethal (2 × MBC) and sublethal (1/4 × MIC) biocide concentrations in 10 ml at 37°C under constant shaking at 200 rev min^−1^, with 500 μl aliquots taken before biocide addition (time 0) and 10 min, 1 and 3 h after biocide addition and diluted with 500 μl of phosphate‐buffered saline (PBS; Thermo Fisher Scientific). ROS was quantified using the CM‐H_2_DCFDA General Oxidative Stress Indicator (C6827, Thermo Fisher Scientific) according to the manufacturer's instructions with CM‐H_2_DCFDA stained cells quantified by flow cytometry (BD LSR II Flow Cytometry, BD Biosciences) with the lower threshold set using the negative and unstained controls, and the upper threshold set using the positive control (Hydrogen Peroxide [H_2_O_2_] at 100 mM). The ROS level was recorded as a percentage on a scale between the lower threshold (0%) and the upper threshold (100%). The test was conducted on three replicate test‐samples with technical triplicates.

### Statistical methods

The antimicrobial efficacy of SAQAS‐A treated surfaces was determined using the Area Under Curve (AUC) determined for each replicate. Data that passed the D'Agostino & Pearson normality test was subjected to a paired t‐test comparing AUC values for each ‘control’ versus the relevant ‘test’ treatment. These tests were deemed to show a significant difference with a *p*‐value of less than 0.05. AUC calculations and statistical tests were conducted in GraphPad Prism version 7.03.

## RESULTS

### Is the SAQAS‐A biocide antimicrobial on treated surfaces for wet and dry inocula?

**FIGURE 1:**
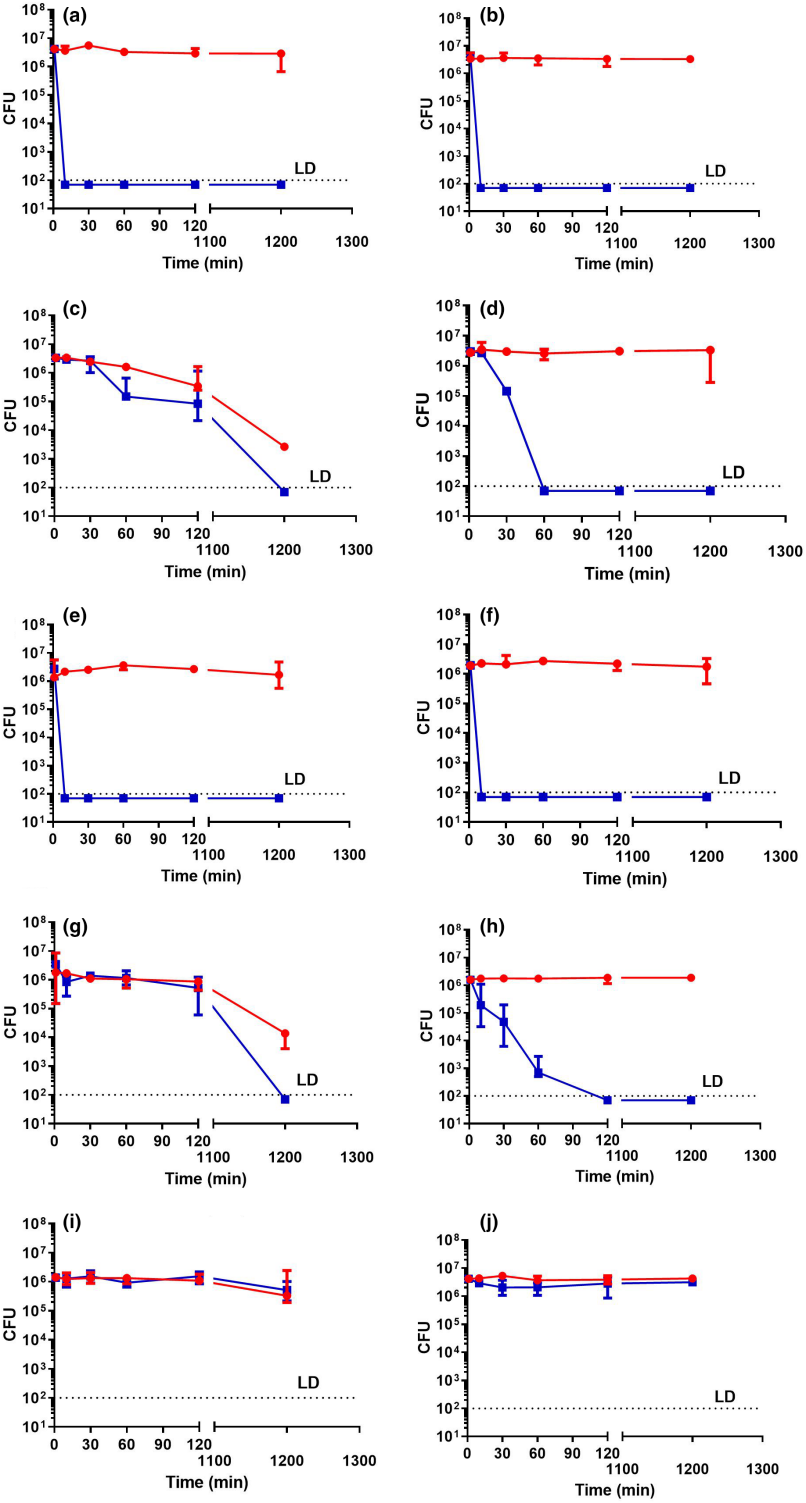
The antimicrobial potency of SAQAS‐A treated glass and LDPE surfaces using a wet fomite test. Untreated (red lines) and SAQAS‐A treated (blue lines) glass (left panel) and LDPE (right panel) surfaces were incubated with 10^6^ CFU of *Staphylococcus aureus* (a, b), *Escherichia coli* (c, d), MRSA (e, f), ESBL *E. coli* (g, h) and *Bacillus cereus* spores (i, j). At various time points, samples were eluted with Letheen Broth and any viable bacteria enumerated by plate counting. Each point is the median of medians for three biological replicates. The range of values is given by the error bar. Abbreviations: CFU, colony forming units; LD, limits of detection.

The antimicrobial activity of a representative Si‐QAC‐type biocide (Daood & Yiu, 2018; Gong et al., 2012) (SAQAS‐A) was first tested against antibiotic‐sensitive strains of *Staphylococcus aureus* (ATCC 6538) and *E. coli* (ATCC 25922) on glass surfaces in wet fomite assays (Figure 1a,c), with bacterial survival determined at five different time points. For *S. aureus*, a clear 4‐log reduction in viability was observed after 10 min incubation with SAQAS‐A while bacteria were recovered from the untreated glass control surface without a noticeable decline in viability. By contrast, a 4‐log reduction in viable *E. coli* compared to the inoculum was only seen after 20 h incubation, and this was mirrored by a decline in viability on the untreated control surface that gave a 3‐log reduction in viability at 20 h.

To investigate if the surface made a difference, the antimicrobial treatment was applied to an LDPE surface, and the survivability of *S. aureus* and *E. coli* was measured (Figure 1b,d). A clear 4‐log reduction of both *S. aureus* and *E. coli* recovery from the treated surface was observed, but without a background of declining viability for *E. coli*, suggesting that some kill of *E. coli* is due to the glass surface. The kill rate for *S. aureus* was more rapid, with a 4‐log reduction seen in 10 min compared to 1 h for *E. coli*.

To investigate if the surfaces would be equally effective against antibiotic‐resistant bacteria, treated glass and LDPE surfaces were challenged with an MRSA strain and an ESBL *E. coli* strain (Figure 1e–h). The kill profiles on treated and untreated surfaces were similar when comparing antibiotic‐sensitive and antibiotic‐resistant isolates of the same species. The only notable difference was the time to a 4‐log reduction in viability for the ESBL *E. coli* increased from 1 to 2 h contact time on the treated LDPE surface.

Previous research has suggested that SAQAS treated surfaces would not be active against bacterial endospores (Bloomfield & Arthur, 1994; Russell, 1990), a limitation for present‐day hospital surfaces where transmission of *Clostridioides difficile* is a concern. We confirmed this inactivity against bacterial endospores using spores isolated from the aerobic model species *B. cereus* (Figure 1i,j).

**FIGURE 2:**
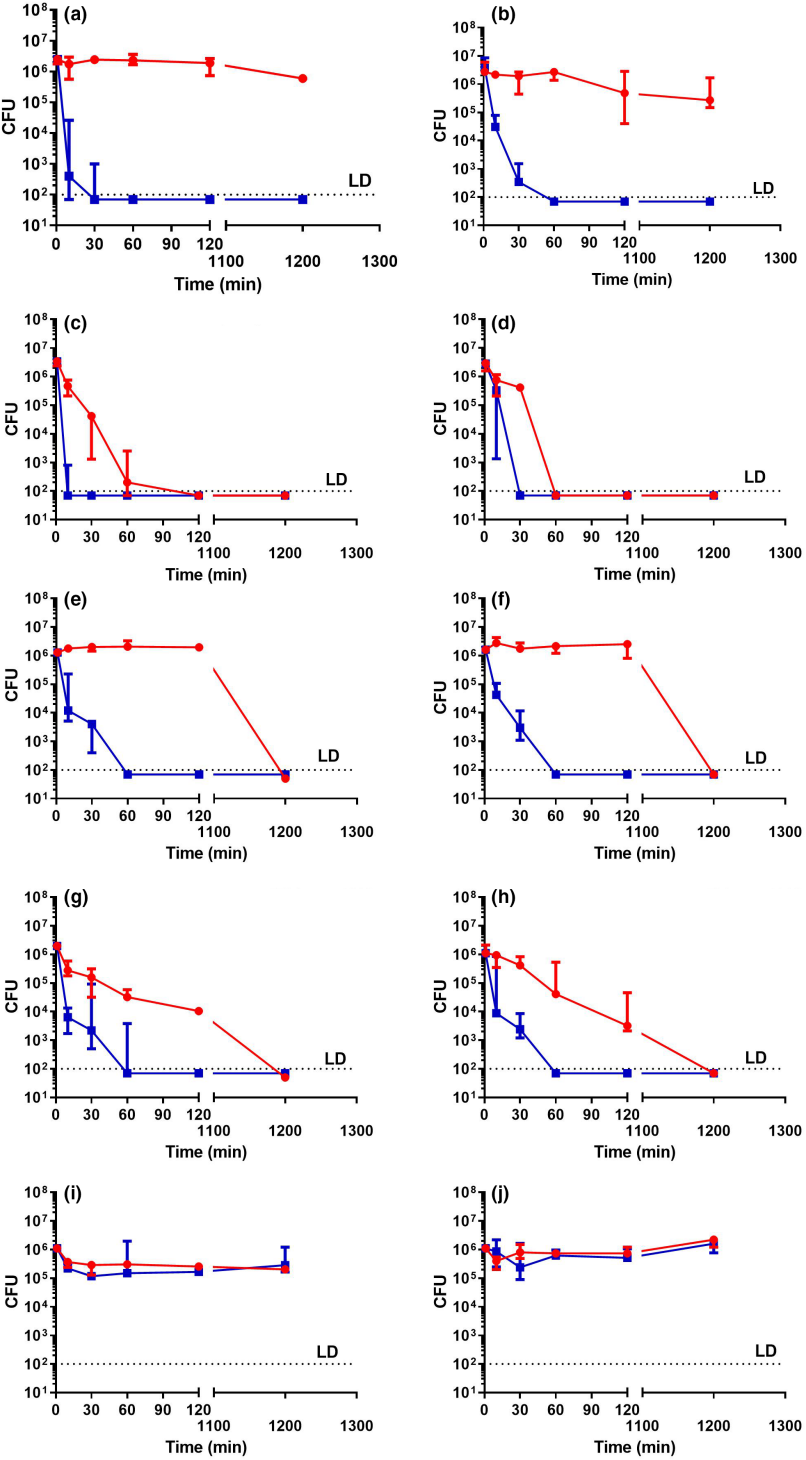
The antimicrobial potency of SAQAS‐A treated glass and plastic surfaces using a dry fomite test. Untreated (red lines) and SAQAS‐A treated (blue lines) glass (left panel) and LDPE (right panel) surfaces were challenged with 10^6^ CFU of *Staphylococcus aureus* (a, b), *E. coli* (c, d), MRSA (e, f), ESBL *E. coli* (g, h) and *B. cereus* spores (i, j). At various time points, samples were eluted with Letheen Broth and any viable bacteria were enumerated by plate counting. Each point is the median of medians for three biological replicates. The range of values is given by the error bar. Abbreviations: CFU, colony forming units; LD, limits of detection.

The wet fomite model is a well‐established test represented by JIS Z 2801:2010, ISO 22196:2011; however, it is limited when considering the conditions many bacteria involved in surface‐mediated transmission are exposed to where a dry fomite model could be more applicable. The antimicrobial activity of SAQAS‐treated and untreated glass and LDPE surfaces was tested against antibiotic‐sensitive and antibiotic‐resistant strains of *S. aureus* and *E. coli* and spores of *B. cereus* in dry fomite assays (Figure 2). Overall the results showed similar trends to the wet fomite assays, but the reduction in viability on untreated surfaces was more apparent, and killing of *S. aureus* on treated surfaces was slower. Area under the curve (AUC) calculations were made to allow analyses of the relative time‐to‐kill on treated and untreated surfaces. The results for wet and dry fomite assays are summarized in Tables 1 and 2, and Tables S1–S4.

**TABLE 1:** Antibacterial activity of SAQAS‐A in wet and dry fomite tests: Time to see a 4‐log reduction in viability

Surface	*Staphylococcus aureus* 6538	MRSA	*Escherichia coli* 25922	ESBL *E. coli*	*Bacillus cereus* spores
Wet Fomite					
SAQAS‐A treated					
Glass	10 min	10 min	20 h	20 h	No killing
LDPE	10 min	10 min	1 h	2 h	No killing
Dry Fomite					
SAQAS‐A treated					
Glass	30 min	1 h	10 min	1 h	No killing
LDPE	30 min	1 h	30 min	1 h	No killing

**TABLE 2:** Antibacterial activity of SAQAS‐A in wet and dry fomite tests: Area under the curve comparisons

	Glass			LDPE		
	SAQAS‐A treated	Untreated	Significance (*p*‐value)	SAQAS‐A treated	Untreated	Significance (*p*‐value)
*Wet fomite*						
*Staphylococcus aureus* 6538	2.02 × 10^6^ ± 1.34 × 10^5^	1.94 × 10^7^ ± 3.16 × 10^6^	<0.0001	2.03 × 10^6^ ± 5.71 × 10^5^	1.73 × 10^7^ ± 2.43 × 10^6^	<0.0001
MRSA	1.37 × 10^6^ ± 1.10 × 10^6^	1.37 × 10^7^ ± 1.98 × 10^6^	<0.0001	9.66 × 10^5^ ± 3.12 × 10^4^	1.14 × 10^7^ ± 2.18 × 10^6^	<0.0001
*E. coli* 25922	8.81 × 10^6^ ± 2.18 × 10^6^	9.99 × 10^6^ ± 1.58 × 10^6^	0.2981	4.69 × 10^6^ ± 1.60 × 10^6^	1.62 × 10^7^ ± 2.46 × 10^6^	<0.0001
ESBL *E. coli*	5.78 × 10^6^ ± 2.96 × 10^6^	6.24 × 10^6^ ± 2.52 × 10^6^	0.4058	1.25 × 10^6^ ± 6.25 × 10^5^	9.28 × 10^6^ ± 1.80 × 10^6^	<0.0001
Dry fomite						
*Staphylococcus aureus* 6538	1.26 × 10^6^ ± 3.30 × 10^5^	1.01 × 10^7^ ± 3.12 × 10^6^	<0.0001	1.93 × 10^6^ ± 8.01 × 10^5^	9.67 × 10^6^ ± 2.94 × 10^6^	0.0002
MRSA	7.51 × 10^5^ ± 1.61 × 10^5^	9.51 × 10^6^ ± 1.38 × 10^6^	<0.0001	1.14 × 10^6^ ± 7.50 × 10^5^	1.05 × 10^7^ ± 3.34 × 10^6^	<0.0001
*E. coli* 25922	1.62 × 10^6^ ± 3.63 × 10^5^	2.14 × 10^6^ ± 3.85 × 10^5^	0.0006	1.30 × 10^6^ ± 1.54 × 10^5^	1.93 × 10^6^ ± 8.63 × 10^5^	0.0514
ESBL *E. coli*	1.15 × 10^6^ ± 1.93 × 10^5^	2.46 × 10^6^ ± 1.36 × 10^6^	0.0117	9.56 × 10^5^ ± 6.68 × 10^5^	2.22 × 10^6^ ± 4.92 × 10^5^	0.0001

*Notes*: Values given are the Area Under Curve (AUC) ± standard deviation, with significance given as the P value obtained. The data passed the D'Agostino & Pearson normality test and was subjected to a paired t‐test comparing the AUC values of treated surfaces with the untreated control surface. A significant reduction is assigned for those comparisons where *p* < 0.05. The data is presented in Tables S1–S4.

The in vitro wet and dry fomite tests used here demonstrate the antimicrobial potency of SAQAS‐A treated surfaces against representative Gram‐positive and Gram‐negative isolates, including antibiotic‐resistant pathogens.

### Mechanism of action for SAQAS‐A against *E. coli* and *S. aureus*

The mechanism of killing attributed to the SAQAS‐type biocides is widely hypothesized to be due to physical puncture of the bacterial membrane leading to cell lysis (McDonnell & Russell, 1999; Oblak et al., 2019). An alternative mechanism involving membrane damage through an ion‐exchange mechanism has been proposed (Crismaru et al., 2011). To investigate the mechanism further, we determined the MIC of the SAQAS‐A in suspension for *E. coli* and *S. aureus*. We examined cells treated with sublethal (0.25 × MIC) and lethal (2 × MIC) levels of SAQAS‐A for membrane damage over time by quantifying: (a) depolarization of the proton gradient; (b) the release of a small molecule (ATP), indicative of small puncture holes, and (c) the release of a larger molecule (DNA), indicative of large holes and lysis. We asked whether this membrane damage correlated with an alternate route to cell‐killing in ROS production and aligned the timing of each membrane damage parameter with the lethal period, measured by the recovery of viable cells on an agar plate. To evaluate any differences between SAQAS‐A challenges in suspension and on a treated surface, we repeated the measurements for bacteria applied to treated surfaces.

**FIGURE 3:**
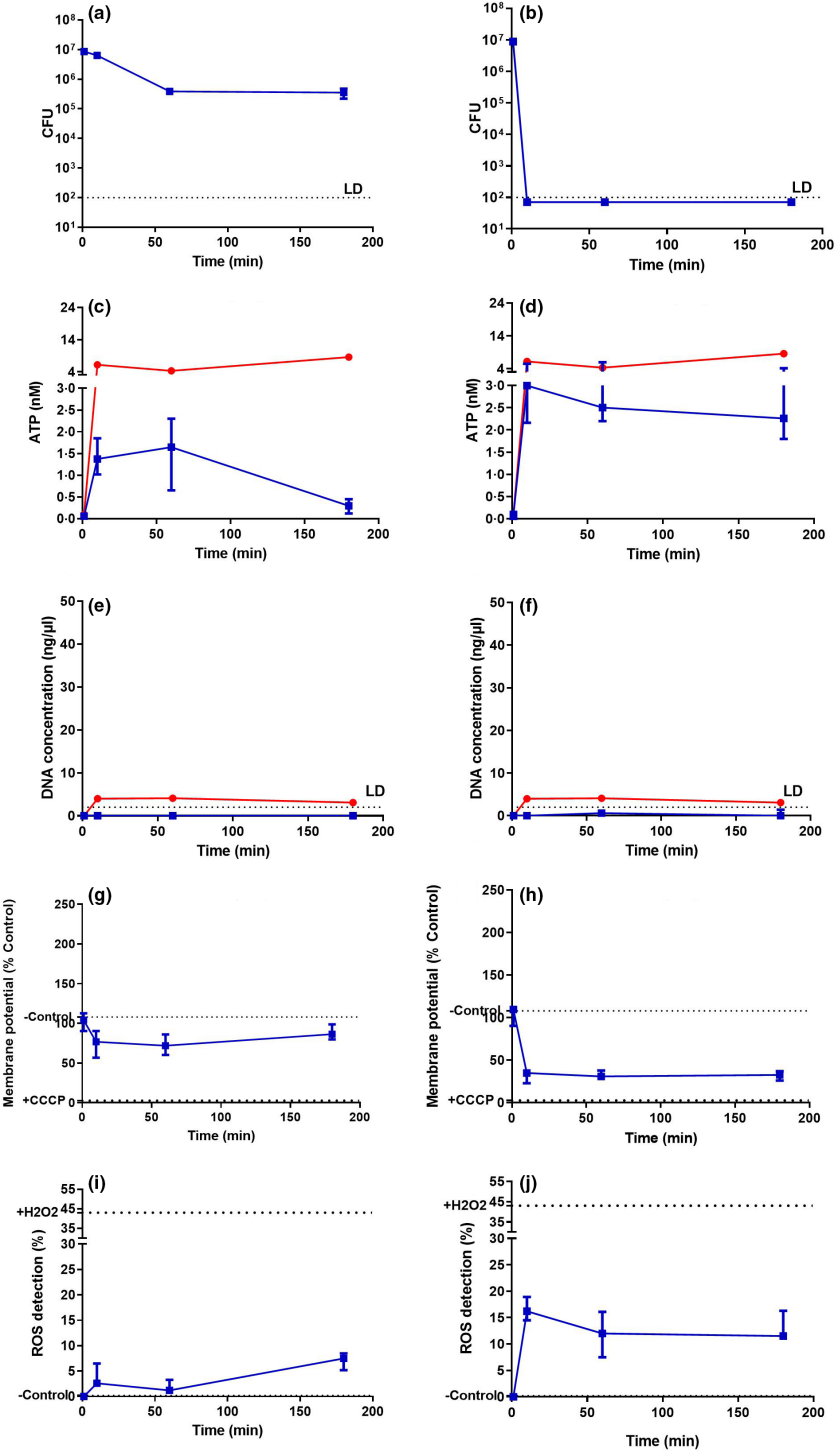
Analyses of the mechanism of action for SAQAS in suspension against *Staphylococcus aureus*. Measurements are taken after 0, 10 min, 1 h and 3 h incubations of cell viability (CFU recovery; a, b); ATP release (c, d); DNA release (e, f); changes in membrane potential (g, h); and ROS production (i, j) for sublethal (a, c, e, g, i) and lethal (b, d, f, h, j) concentrations. Each point is the median of medians for three biological replicates. The range of values is given by the error bar. Abbreviation: LD, limits of detection. The red line represents polymyxin‐B, and the blue line represents SAQAS‐A.

**FIGURE 4:**
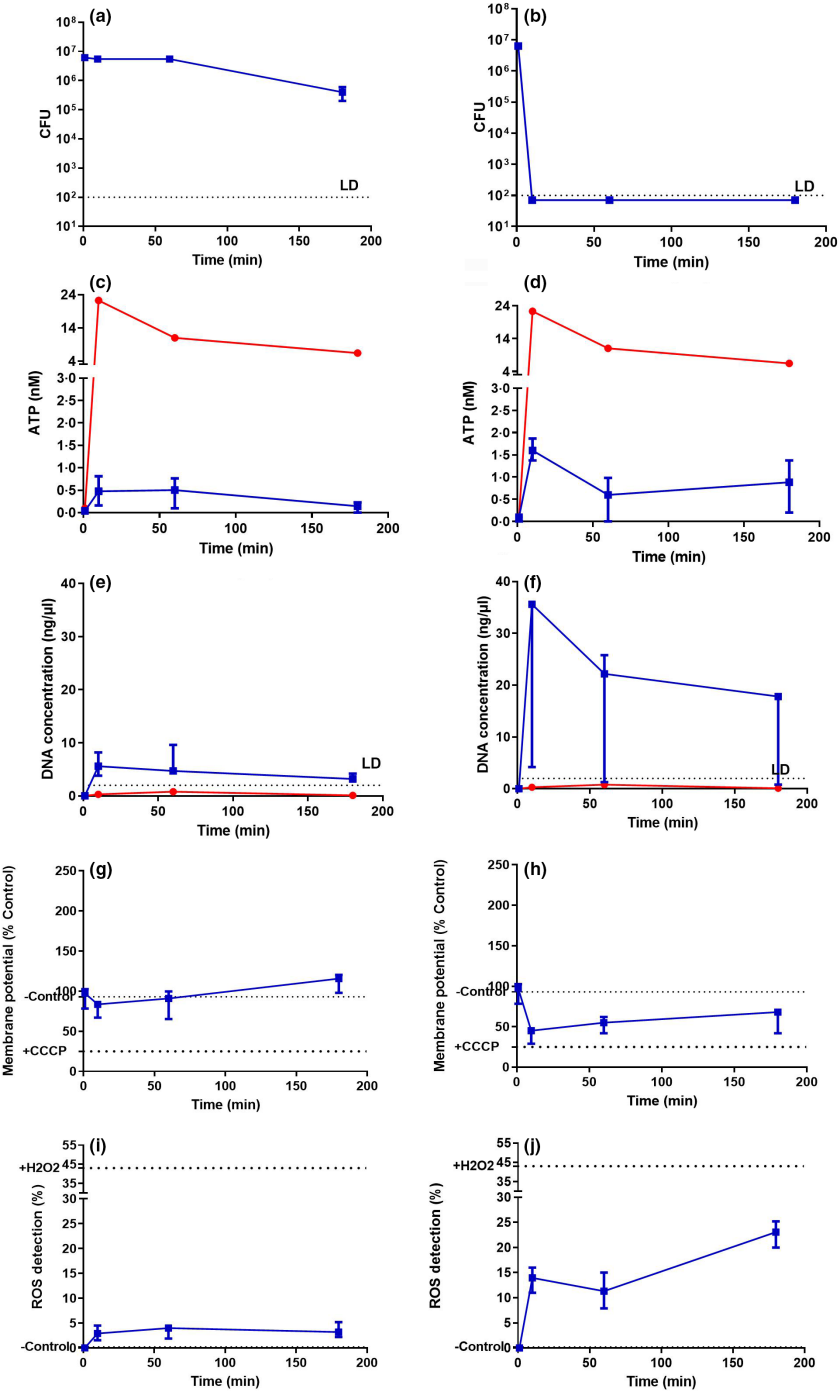
Analyses of the mechanism of action for SAQAS in suspension against *Escherichia coli*. Measurements are taken after 0, 10 min, 1 and 3 h incubations of cell viability (CFU recovery; a, b); ATP release (c, d); DNA release (e, f); changes in membrane potential (g, h); and ROS production (i, j) for sublethal (a, c, e, g, i) and lethal (b, d, f, h, j) concentrations. Each point is the median of medians for three biological replicates. The range of values is given by the error bar. Key: LD, limits of detection. The red line represents polymyxin‐B, and the blue line represents SAQAS‐A.

For *S. aureus* (Figure 3), SAQAS‐A in suspension gave a 10^6^‐fold reduction in viability in 10 min (Figure 3b) at 2 × MIC, but only around a 10× reduction in viable cells at 0.25 × MIC over a 3 h challenge (Figure 3a). Correlating with lethal membrane damage, an initial peak of ATP release was equivalent to that seen with polymyxin B, which is known to form pores in the membrane, allowing the complete release of cellular ATP (Ferrari et al., 2004; Gales et al., 2001; Zavascki et al., 2007). At the same time (within 10 min), there was membrane depolarization accompanied by ROS production but no DNA release. At sublethal levels of SAQAS‐A ATP release, membrane depolarization and ROS production were observed, although not to the extent seen in the lethal treatment, and these returned to control levels over the period between 10 min and 3 h. Similar results were obtained with challenges of *E. coli* (Figure 4), with the exception that substantial DNA release, indicating cell lysis, was seen at 10 min.

**FIGURE 5:**
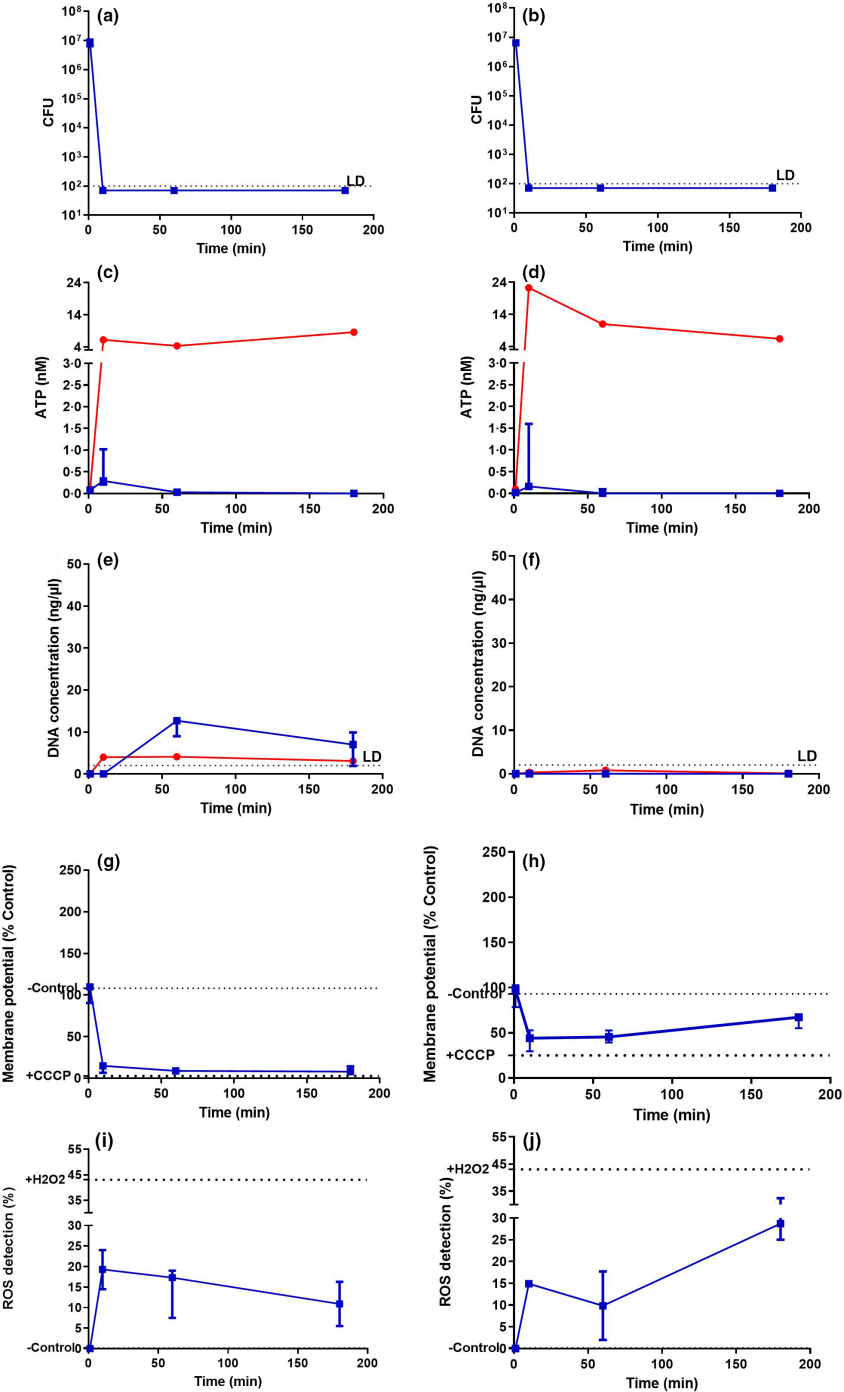
Analyses of the mechanism of action for SAQAS‐A treated surfaces against *Staphylococcus aureus* and *Escherichia coli*. Measurements are taken after 0, 10 min, 1 and 3 h incubations of cell viability (CFU recovery; a, b); ATP release (c, d); DNA release (e, f); changes in membrane potential (g, h); and ROS production (i, j) for *S. aureus* (a, c, e, g, i) and *E. coli* (b, d, f, h, j). Each point is the median of medians for three biological replicates. The range of values is given by the error bar. Key: LD, limits of detection. The red line represents polymyxin‐B, and the blue line represents SAQAS‐A.

By comparison, the challenge of *S. aureus* and *E. coli* on contact with SAQAS‐A treated surfaces, while lethal after 10 min, resulted in a smaller peak of ATP release, but DNA release from *S. aureus* suggesting lysis and membrane depolarization accompanied by ROS production (Figure 5).

Overall, the results show a difference in assay results between sublethal and lethal SAQAS‐A treatments in suspension, highlighting the correlation of small membrane punctures, membrane depolarization and ROS generation with bacterial death. For *E. coli* in suspension, membrane damage progressed to cell lysis (Figure 4). For treatments at a surface, measurement suggests a greater role for membrane depolarization and ROS generation (Figure 5).

## DISCUSSION

Healthcare‐associated infections (HAI) caused by fomite‐mediated transmission are significant concerns in hospitals, associated with human and economic costs (Desai et al., 2016; Reed & Kemmerly, 2009). A possible solution for preventing fomite‐mediated transmission is using antimicrobial coatings with long‐lasting activity. Here we have tested a commercially available contact‐dependent biocide formulation based upon the active siloxane anchored quaternary ammonium salt (SAQAS) and show promising activity against representative species of Gram‐positive (*S. aureus*) and Gram‐negative (*E. coli*) pathogens, including antibiotic‐resistant strains, but no activity against endospores of *B. cereus*.

Standard methods are required for both industry and regulators to test the effectiveness of novel products that claim to have antimicrobial activity. There is no single test method that evaluates the efficacy of antimicrobial surface designs, as the method used may vary based on the mechanism of action and the properties of the surface material. The JIS Z 2801 and ISO 22196 protocols represent a commonly cited standard assay for checking the antimicrobial potency of a surface incorporated with a biocide (Hauser & Wunderlich, 2011; Mathew et al., 2020; Michels et al., 2009). Here we have used the assay protocol to check the antimicrobial potency of SAQAS biocides in wet conditions and adapted the inoculation protocol to test biocides in dry conditions. The assays test the activity of biocidal surfaces inoculated with TSB‐grown bacteria diluted in saline, although more information may be obtained inoculating in the presence of proteinaceous soil and using biofilm inocula. Viable cells were recovered from the surface using Letheen Broth (Engelbrecht et al., 2013; Mackinnon, 1974; Springthorpe & Sattar, 2007) to neutralize biocide leaching from the surface. The commercial product (SAQAS‐A) was used at room temperature to mimic real‐world conditions.

The antimicrobial potency of SAQAS‐A was higher towards *S. aureus* than *E. coli* in both wet and dry conditions. This suggests that SAQAS‐A‐treated surfaces may be more effective against Gram‐positive bacteria than Gram‐negative. However, this would need to be tested using a larger number of species. Gram‐positive bacteria are considered to survive better on dry surfaces (Galvin et al., 2012) than Gram‐negative bacteria (Dominguez‐Wong et al., 2014), which further supports our findings in dry fomite tests using treated surfaces (Table 1). Importantly, the antibiotic resistance profiles of the strains tested in this study did not affect the sensitivity of *S. aureus* and *E. coli* to SAQAS‐A‐treated surfaces. While our data shows that SAQAS‐A‐treated surfaces were active against vegetative bacteria, they were not active against endospores of *B. cereus*. This suggests that SAQAS‐treated surfaces are unlikely to have any activity against the endospores of the critical hospital pathogen *C. difficile* (Mazuski et al., 2019; Mutters et al., 2009). Thus, there is room to improve SAQAS‐based coatings for application in the real world, especially in healthcare facilities.

In this study, SAQAS‐A was applied to LDPE or glass carriers, with little difference in the antimicrobial potency. However, *E. coli* experienced a loss in viability on untreated glass surfaces and in dry conditions that is important to control for. The study is limited by only testing these two surfaces, and for completeness, checking effectiveness on all materials that might be protected by these products would be ideal. In addition, future studies should consider, in detail, the nature of the surface and the anchoring chemistry that underpins the manufacturers' representations of long‐lasting activity for SAQAS‐biocides. For example, the LDPE carriers used in this study would not be expected to present hydroxy groups for covalent attachment, so we are left to question if the active Si‐QAC agent attaches?

Investigating the mode of action of the SAQAS against both Gram‐positive and Gram‐negative bacteria gave findings that support a hypothesis of membrane damage leading to cell death. Nevertheless, while the SAQAS‐A disinfectant in suspension seemed to only puncture small holes in the *S. aureus* membrane, as ATP but not DNA release was observed, it was able to puncture larger holes in *E. coli* and disrupt membrane integrity, releasing large nucleic acid molecules and small ATP molecules upon cell death. By contrast, the surface anchored‐SAQAS‐A was able to puncture large holes in *S. aureus*, releasing DNA upon cell death, but only puncture small holes in *E. coli*. During the initial 10 min time interval, there was a substantial decrease in membrane potential in all SAQAS‐A experimental samples, with a surge in ROS present in the samples, potentially aiding in cell death (Kohanski et al., 2007). These results support SAQAS‐A lethality against both Gram‐positive *S. aureus* and Gram‐negative *E. coli*, requiring simple puncture of cells leading to a loss of small molecules and membrane depolarization with ROS production. Future work could examine the sequence of these events in more detail.

**FIGURE 6:**
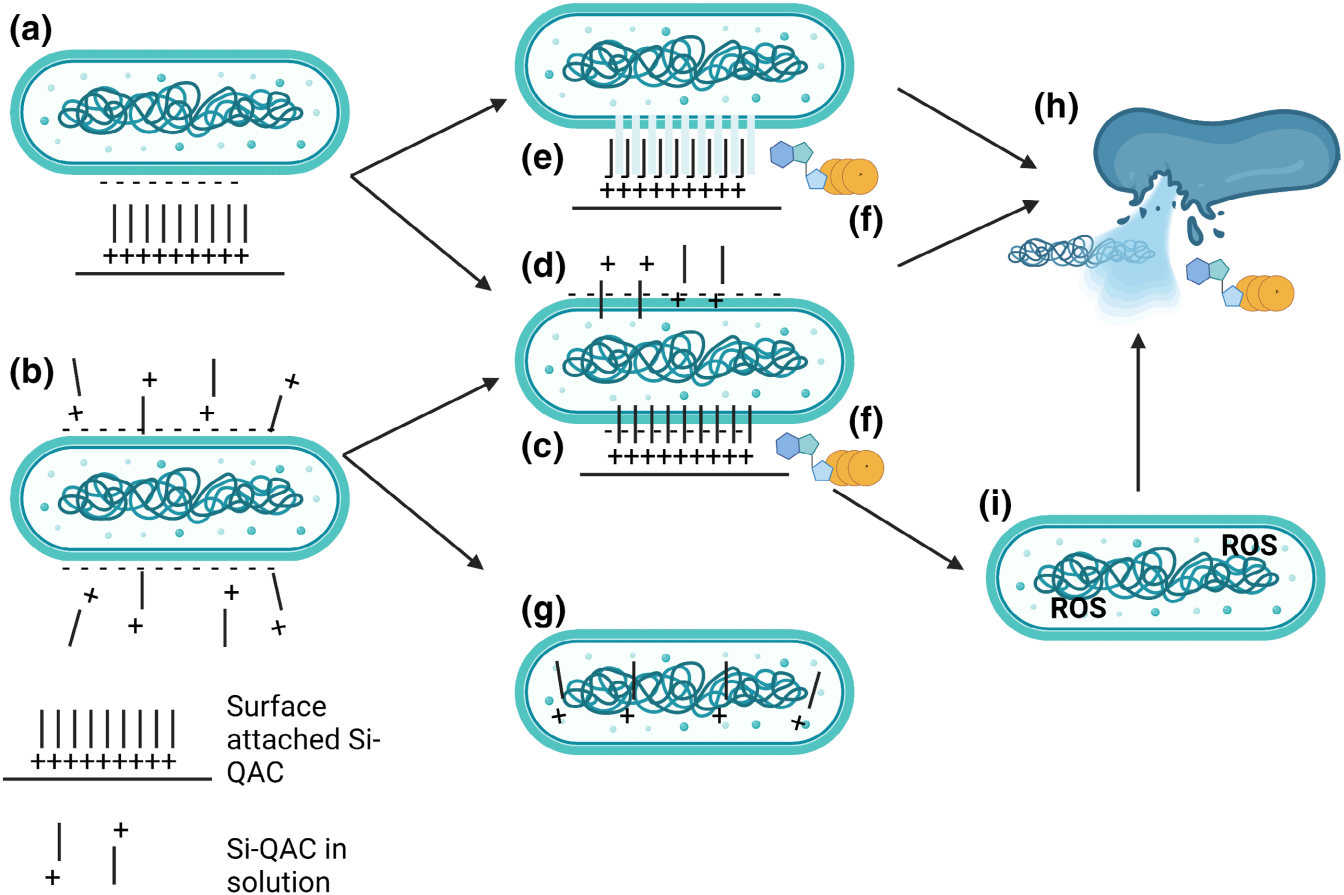
Antimicrobial mechanisms of SAQAS action. The potential antibacterial mechanisms of action of SAQAS disinfectant attached to surfaces (a) and in solution (b) are depicted. Electrostatic attraction of the positively charged quaternary ammonium Nitrogen to the negative charged bacterium may puncture cell membranes (c), lead to QAC‐mediated ion exchange and reactions that damage the bacterial cell envelope (d) or promote the suctioning of membrane that damages the bacterial envelope (e). The small holes produced in process c, d and e lead to nondestructive membrane disturbances including the release of ATP and other small molecules (f) and may lead to membrane depolarization. The SAQAS in solution may penetrate the cell and cause lethal QAC‐mediated damage to intracellular components (g). Membrane depolarization or other mechanisms may lead to an uncoupling of electron transport from ATP synthesis and the intracellular production of lethal reactive oxygen species (ROS; i). Envelope damage from physical puncture and/or chemical damage from QAC or ROS may then lead to destructive membrane disturbances and cell lysis, with the release of larger molecules like DNA (h). Figure created with BioRender.com.

The SAQAS biocides can be described as cationic antimicrobial polymers, for which there have been detailed investigations of possible mechanisms of action. A recent review of the field categorized mechanisms that were (i) nondestructive membrane disturbances, (ii) destructive disturbances or (iii) intracellular (Yu et al., 2022). Our suspension studies at sub‐MIC concentrations suggest nondestructive membrane disturbances (e.g. membrane depolarization and small holes that allow ATP release) from which cells may recover. At higher lethal concentrations, nondestructive membrane disturbances still occur, and may progress to destructive disturbances characterized by cell lysis and the release of DNA. Intracellular contributions to killing may include the penetration of Si‐QAC from solutions into cells to act as QACs and the generation of ROS, perhaps as a result of nondestructive membrane disturbances. The dogma associated with SAQAS coatings suggests a ‘puncture’ mechanism with many small holes, consistent with the loss of ATP and membrane depolarization, but not DNA. A mechanism with more extensive membrane damage may see the coalescence of these small holes, or as has been proposed by computer simulations, the suctioning out of parts of the anionic bacterial membrane (Li et al., 2011). A summary of the possible antibacterial mechanisms of SAQAS is given in Figure 6. A stronger appreciation of the details of the mechanism of action may be realized by the application of techniques such as super‐resolution STimulated Emission Depletion (STED) microscopy, cryo Electron Microscopy and Atomic Force Microscopy (Yu et al., 2022), and should further attempt to differentiate between biocide that is solely surface anchored and biocide in solution.

Finally, these in vitro tests are conducted for a single challenge on treated surfaces; however, in real life, the surface needs to retain activity after multiple consecutive challenges and with routine cleaning and disinfection. The ultimate aim is to apply SAQAS on frequently touched surfaces in the hospital. This means that any treatment needs to work on a surface for an extended period and against varied inocula in terms of the species and numbers of cells. Future studies should investigate the efficacy of SAQAS‐treated surfaces in work that incorporates both real‐world testing of the protection offered on different surface types and repetition of the activity testing we report here on surfaces to evaluate stability (do they still work after 30 days?) and durability to disinfection (do they still work after surface disinfection?) and is the Si‐QAC molecule detectable at the surface before and after disinfection?

## CONFLICT OF INTEREST

No conflict of interest is declared.

## Supporting information


Table S1

## References

[jam15729-bib-0001] Ahmed, E.H. , Hassan, H.M. , El‐sherbiny, N.M. and Soliman, A.M.A. (2019) Bacteriological monitoring of inanimate surfaces and equipment in some referral hospitals in Assiut City, Egypt, International Journal of Microbiology [Preprint]. doi:10.1155/2019/5907507, 2019, 1, 9. PMC6745147 31565058

[jam15729-bib-0002] Alfa, M.J. (2019) Biofilms on instruments and environmental surfaces: do they interfere with instrument reprocessing and surface disinfection? Review of the literature. American Journal of Infection Control, 47, A39–A45. 10.1016/j.ajic.2019.02.027 31146849

[jam15729-bib-0003] Allegranzi, B. , Nejad, S.B. , Combescure, C. , Graafmans, W. , Attar, H. , Donaldson, L. et al. (2011) Burden of endemic health‐care‐associated infection in developing countries: systematic review and meta‐analysis. Lancet, 377, 228–241. 10.1016/S0140-6736(10)61458-4 21146207

[jam15729-bib-0004] Andersen, E.V.M. (2016) Bacterial resistance to biocides. In: Handbook of hygiene control in the food industry. Cambridge, UK: Woodhead Publishing, pp. 603–616. 10.1016/B978-0-08-100155-4.00039-X

[jam15729-bib-0005] Arefian, H. , Hagel, S. , Diplwirt‐inf, S.H. , Dipllnf, F.R. , Scherag, A. , Brunkhorst, F.M. et al. (2016) Extra length of stay and costs because of health care associated infections at a German university hospital. American Journal of Infection Control, 44, 160–166. 10.1016/j.ajic.2015.09.005 26521700

[jam15729-bib-0006] ASTM Standard . (2012) Standard test method for applying aerosolized Bacillus spores as dry inocula to inanimate surfaces. United States: ASTM International. 10.1520/E2894-12.2

[jam15729-bib-0007] Bloomfield, S.F. & Arthur, M. (1994) Mechanisms of inactivation and resistance of spores to chemical biocides. The Journal of Applied Bacteriology, 76, 91S–104S. 10.1111/j.1365-2672.1994.tb04361.x 8047915

[jam15729-bib-0008] Bures, S. , Fishbain, J.T. , Uyehara, C.F.T. , Parker, J.M. & Berg, B.W. (2000) Computer keyboards and faucet handles as reservoirs of nosocomial pathogens in the intensive care unit. American Journal of Infection Control, 28(6), 465–471. 10.1067/mic.2000.107267 11114617

[jam15729-bib-0009] Chapman, J.S. (2003) Disinfectant resistance mechanisms, cross‐resistance, and co‐resistance. International Biodeterioration & Biodegradation, 51(4), 271–276. 10.1016/S0964-8305(03)00044-1

[jam15729-bib-0010] Crismaru, M. , Asri, L.A.T.W. , Loontjens, T.J.A. , Krom, B.P. , De Vries, J. , Van Der Mei, H.C. et al. (2011) Survival of adhering staphylococci during exposure to a quaternary ammonium compound evaluated by using atomic force microscopy imaging. Antimicrobial Agents and Chemotherapy, 55(11), 5010–5017. 10.1128/aac.05062-11 21876063 PMC3195039

[jam15729-bib-0011] Dancer, S.J. (2004) How do we assess hospital cleaning? A proposal for microbiological standards for surface hygiene in hospitals. Journal of Hospital Infection, 56, 10–15. 10.1016/j.jhin.2003.09.017 14706265 PMC7134512

[jam15729-bib-0012] Daood, U. & Yiu, C.K.Y. (2018) Transdentinal cytotoxicity and macrophage phenotype of a novel quaternary ammonium silane cavity disinfectant. Dental Materials, 35(2), 206–216. 10.1016/j.dental.2018.11.018 30509480

[jam15729-bib-0013] Desai, K. , Gupta, S.B. , Dubberke, E.R. , Prabhu, V.S. , Browne, C. & Mast, T.C. (2016) Epidemiological and economic burden of *Clostridium difficile* in the United States: estimates from a modeling approach. BMC Infectious Disease, 16(303), 16. 10.1186/s12879-016-1610-3 PMC4912810 27316794

[jam15729-bib-0014] Dominguez‐Wong, C. , Loredo‐Becerra, G.M. , Quintero‐González, C.C. , Noriega‐Treviño, M.E. , Compeán‐Jasso, M.E. , Niño‐Martínez, N. et al. (2014) Evaluation of the antibacterial activity of an indoor waterborne architectural coating containing Ag/TiO_2_ under different relative humidity environments. Materials Letters, 134, 103–106. 10.1016/j.matlet.2014.07.067

[jam15729-bib-0015] Dwyer, D.J. , Belenky, P.A. , Yang, J.H. & Macdonald, I.C. (2014) Antibiotics induce redox‐related physiological alterations as part of their lethality. Proceedings of the National Academy of Sciences, 111, E2100–E2109. 10.1073/pnas.1401876111 PMC4034191 24803433

[jam15729-bib-0016] Engelbrecht, K. , Ambrose, D. , Sifuentes, L. , Gerba, C. , Weart, I. & Koenig, D. (2013) Decreased activity of commercially available disinfectants containing quaternary ammonium compounds when exposed to cotton towels. American Journal of Infection Control, 41(10), 908–911. 10.1016/j.ajic.2013.01.017 23623007

[jam15729-bib-0017] Ferrari, D. , Pizzirani, C. , Adinolfi, E. , Forchap, S. , Sitta, B. , Turchet, L. et al. (2004) The antibiotic polymyxin B modulates P2X 7 receptor function. Journal of Immunology, 173(7), 4652–4660. 10.4049/jimmunol.173.7.4652 15383600

[jam15729-bib-0019] Gales, A.C. , Reis, A.O. & Jones, R.N. (2001) Contemporary assessment of antimicrobial susceptibility testing methods for polymyxin B and colistin: Review of available interpretative criteria and quality control guidelines. Journal of Clinical Microbiology, 39(1), 183–190. 10.1128/JCM.39.1.183-190.2001 11136768 PMC87699

[jam15729-bib-0020] Galvin, S. , Dolan, A. , Cahill, O. , Daniels, S. & Humphreys, H. (2012) Microbial monitoring of the hospital environment: why and how? The Journal of Hospital Infection, 82(3), 143–151. 10.1016/j.jhin.2012.06.015 23022372

[jam15729-bib-0021] Ghosh, S. & Haldar, J. (2020) Cationic polymer–based antibacterial smart coatings. In: Advances in smart coatings and thin films. Amsterdam, Netherlands: Elsevier Inc, pp. 557–582. 10.1016/b978-0-12-849870-5.00011-2

[jam15729-bib-0022] Gong, S.Q. , Niu, L.N. , Kemp, L.K. , Yiu, C.K.Y. , Ryou, H. , Qi, Y.P. et al. (2012) Quaternary ammonium silane‐functionalized, methacrylate resin composition with antimicrobial activities and self‐repair potential. Acta Biomaterialia, 8(9), 3270–3282. 10.1016/j.actbio.2012.05.031 22659173 PMC3580770

[jam15729-bib-0023] Graves, N. , Nicholls, T.M. , Christopher, G.S. & Morris, A.J. (2003) The prevalance and estimates of the cumulative incidence of hospital acquired infections among patients admitted to Auckland District Health Board hospitals in New Zealand. Infection Control and Hospital Epidemiology, 24(1), 56–61. 12558237 10.1086/502116

[jam15729-bib-0024] Hartmann, M. , Berditsch, M. , Hawecker, J. , Ardakani, M.F. , Gerthsen, D. & Ulrich, A.S. (2010) Damage of the bacterial cell envelope by antimicrobial peptides gramicidin S and PGLa as revealed by transmission and scanning electron microscopy. Antimicrobial Agents and Chemotherapy, 54(8), 3132–3142. 10.1128/AAC.00124-10 20530225 PMC2916356

[jam15729-bib-0025] Hauser, C. & Wunderlich, J. (2011) Antimicrobial packaging films with a sorbic acid based coating. Procedia Food Science, 1, 197–202. 10.1016/j.profoo.2011.09.031

[jam15729-bib-0026] Hota, B. (2004) Contamination, disinfection, and cross colonization: Are hospital surfaces reserviors for nosocomial infections? Clinical Infectious Diseases, 39(8), 1182–1189. 10.1086/424667 15486843 PMC7107941

[jam15729-bib-0027] Ioannou, C.J. , Hanlon, G.W. & Denyer, S.P. (2007) Action of disinfectant quaternary ammonium compounds against *Staphylococcus aureus* . Antimicrobial Agents and Chemotherapy, 51(1), 296–306. 10.1128/AAC.00375-06 17060529 PMC1797692

[jam15729-bib-0028] Isquith, A.J. , Abbott, E.A. & Walters, P.A. (1972) Surface‐bonded antimicrobial activity of an organosilicon quaternary ammonium chloride. Applied Micriobiology, 24(6), 859–863. 10.1128/am.24.6.859-863.1972 PMC380687 4650597

[jam15729-bib-0029] JIS Z 2801 . (2010) Antibacterial products—test for antibacterial activity and efficacy. Japan: Japanese Standard Associaion. https://webdesk.jsa.or.jp/preview/pre_jis_z_02801_000_000_2010_e_ed10_i4.pdf

[jam15729-bib-0030] Kaur, R. & Liu, S. (2016) Antibacterial surface design‐contact kill. Progress in Surface Science, 91(3), 136–153. 10.1016/j.progsurf.2016.09.001

[jam15729-bib-0031] Kohanski, M.A. , Dwyer, D.J. , Hayete, B. & Lawrence, C.A. (2007) A common mechanism of cellular death induced by bactericidal antibiotics. Cell, 130, 797–810. 10.1016/j.cell.2007.06.049 17803904

[jam15729-bib-0032] Kramer, A. , Schwebke, I. & Kampf, G. (2006) How long do nosocomial pathogens persist on inanimate surfaces? A systematic review. BMC Infectious Disease, 6, 130. 10.1186/1471-2334-6-130 PMC1564025 16914034

[jam15729-bib-0033] Langsrud, S. , Sidhu, M.S. , Heir, E. & Holck, A.L. (2003) Bacterial disinfectant resistance a challenge for the food industry. International Biodeterioration & Biodegradation, 51(4), 283–290. 10.1016/S0964-8305(03)00039-8

[jam15729-bib-0034] Legido‐Quigley, C. , Marlin, N.D. , Melin, V. , Manz, A. & Smith, N.W. (2003) Advances in capillary electrochromatography and micro‐high performance liquid chromatography. Electrophoresis, 24(6), 917–944. 10.1002/elps.200390136 12658680

[jam15729-bib-0035] Lemmen, S.W. , Hafner, H. , Zolldann, D. , Stanzel, S. & Lutticken, R. (2004) Distribution of multi‐resistant Gram‐negative versus Gram‐positive bacteria in the hospital inanimate environment. The Journal of Hospital Infection, 56, 191–197. 10.1016/j.jhin.2003.12.004 15003666

[jam15729-bib-0036] Li, H. , Bao, H. , Bok, K.X. , Lee, C. , Li, B. , Zin, M.T. et al. (2016) High durability and low toxicity antimicrobial coatings fabricated by quaternary ammonium silane copolymers. Biomaterials Science, 4, 299–309. 10.1039/c5bm00353a 26535418

[jam15729-bib-0037] Li, P. , Poon, Y.F. , Li, W. , Zhu, H.Y. , Yeap, S.H. , Cao, Y. et al. (2011) A polycationic antimicrobial and biocompatible hydrogel with microbe membrane suctioning ability. Nature Materials, 10(2), 149–156. 10.1038/nmat2915 21151166

[jam15729-bib-0038] Mackinnon, I.H. (1974) The use of inactivators in the evaluation of disinfectants. The Journal of Hygiene, 73(2), 189–195. 10.1017/S0022172400024013 4214496 PMC2130323

[jam15729-bib-0039] Malik, R.E. , Cooper, R.A. & Griffith, C.J. (2003) Use of audit tools to evaluate the efficacy of cleaning systems in hospitals. American Journal of Infection Control, 31(3), 181–187. 10.1067/mic.2003.34 12734526

[jam15729-bib-0040] Mathew, R.T. , Cooney, R.P. , Doyle, C.S. , Swift, S. & Haessler, C. (2020) Anchored quaternary ammonium salts adsorbed on polyurethane film surfaces. Progress in Organic Coating, 138, 105343. 10.1016/j.porgcoat.2019.105343

[jam15729-bib-0041] Mathew, R.T. , Cooney, R.P. , Malmstrom, J. & Doyle, C.S. (2018) Atomic force microscopy and angular‐dependent X‐ray photoelectron spectroscopy studies of anchored quaternary ammonium salt biocides on quartz surfaces. Langmuir, 34(16), 4750–4761. 10.1021/acs.langmuir.8b00535 29597350

[jam15729-bib-0042] Mathew, R.T. , Cooney, R.P. , Zujovic, Z. , Doyle, C. , Wheelwright, W. & de Silva, K. (2018) A sustained release anchored biocide system utilizing the honeycomb cellular structure of expanded perlite. ACS Applied Bio Materials, 1(6), 1959–1971. 10.1021/acsabm.8b00495 34996258

[jam15729-bib-0043] Mazuski, J.E. , Sartelli, M. , Di Bella, S. , Mcfarland, L.V. , Khanna, S. , Furuya‐kanamori, L. et al. (2019) 2019 update of the WSES guidelines for management of Clostridioides (Clostridium) difficile infection in surgical patients. World Journal of Emergency Surgery, 14, 8. 10.1186/s13017-019-0228-3 30858872 PMC6394026

[jam15729-bib-0044] McDonnell, G. & Russell, A.D. (1999) Antiseptics and disinfectants: activity, action, and resistance. Clinical Microbiology Reviews, 12(1), 147–179. 9880479 10.1128/cmr.12.1.147 PMC88911

[jam15729-bib-0045] Michels, H.T. , Noyce, J.O. & Keevil, C.W. (2009) Effects of temperature and humidity on the efficacy of methicillin‐resistant Staphylococcus aureus challenged antimicrobial materials containing silver and copper. Letters in Applied Microbiology, 49(2), 191–195. 10.1111/j.1472-765X.2009.02637.x 19413757 PMC2779462

[jam15729-bib-0046] Mutters, R. , Nonnenmacher, C. , Susin, C. , Albrecht, U. , Kropatsch, R. & Schumacher, S. (2009) Quantitative detection of *Clostridium difficile* in hospital environmental samples by real‐time polymerase chain reaction. The Journal of Hospital Infection, 71(1), 43–48. 10.1016/j.jhin.2008.10.021 19041162

[jam15729-bib-0047] Neely, A.N. & Maley, M.P. (2000) Survival of Enterococci and Staphylococci on hospital fabrics and plastics. Journal of Clinical Microbiology, 38(2), 724–726. 10655374 10.1128/jcm.38.2.724-726.2000 PMC86187

[jam15729-bib-0048] Oblak, E. , Piecuch, A. , Rewak‐Soroczynska, J. & Paluch, E. (2019) Activity of gemini quaternary ammonium salts against microorganisms. Applied Microbiology and Biotechnology, 103, 625–632. 10.1007/s00253-018-9523-2 30460534

[jam15729-bib-0049] Otter, J.A. , Yezli, S. , Salkeld, J.A.G. & French, G.L. (2013) Evidence that contaminated surfaces contribute to the transmission of hospital pathogens and an overview of strategies to address contaminated surfaces in hospital settings. American Journal of Infection Control, 41, S6–S11. 10.1016/j.ajic.2012.12.004 23622751

[jam15729-bib-0050] Read, K. & Bhally, H. (2015) “Real‐time” burden of community and healthcare‐related infections in medical and rehabilitation patients in a public hospital in Auckland, New Zealand. New Zealand Medical Journal, 128(1426), 69–74. 26913909

[jam15729-bib-0051] Reed, D. & Kemmerly, S.A. (2009) Infection control and prevention: a review of hospital‐acquired infections and the economic implications. The Ochsner Journal, 9(1), 27–31. 21603406 PMC3096239

[jam15729-bib-0052] Russell, A.D. (1990) Bacterial spores and chemical sporicidal agents. Clinical Microbiology Reviews, 3(2), 99–119. 2187595 10.1128/cmr.3.2.99 PMC358146

[jam15729-bib-0053] Russotto, V. , Cortegiani, A. , Raineri, S.M. & Giarratano, A. (2015) Bacterial contamination of inanimate surfaces and equipment in the intensive care unit. Journal of Intensive Care, 3, 54. 10.1186/s40560-015-0120-5 26693023 PMC4676153

[jam15729-bib-0054] Sambhy, V. , Peterson, B.R. & Sen, A. (2008) Multifunctional silane polymers for persistent surface derivatization and their antimicrobial properties. Langmuir, 24(14), 7549–7558. 10.1021/la800858z 18547073

[jam15729-bib-0055] Schmidt, M.G. , Attaway, H.H. , Sharpe, P.A. , John, J. , Sepkowitz, K.A. , Morgan, A. et al. (2012) Sustained reduction of microbial burden on common hospital surfaces through introduction of copper. Journal of Clinical Microbiology, 50(7), 2217–2223. 10.1128/JCM.01032-12 22553242 PMC3405627

[jam15729-bib-0056] Shahaby, A.F. , Awad, N.S. , El‐Tarras, A.E. & Bahobial, A.S. (2012) Mobile phone as potential reservoirs of bacterial pathogens. African Journal of Biotechnology, 11(92), 15896–15904. 10.5897/ajb12.1836

[jam15729-bib-0057] Springthorpe, V.S. & Sattar, S.A. (2007) Application of a quantitative carrier test to evaluate microbicides against mycobacteria. Journal of AOAC International, 90(3), 817–824. 17580635

[jam15729-bib-0058] Warnes, S.L. & Keevil, C.W. (2011) Mechanism of copper surface toxicity in vancomycin‐resistant enterococci following wet or dry surface contact. Applied and Environmental Microbiology, 77(17), 6049–6059. 10.1128/AEM.00597-11 21742916 PMC3165410

[jam15729-bib-0059] Weber, D.J. , Rutala, W.A. , Miller, M.B. , Huslage, K. & Sickbert‐Bennett, E. (2010) Role of hospital surfaces in the transmission of emerging health care‐associated pathogens: norovirus, *Clostridium difficile*, and Acinetobacter species. American Journal of Infection Control, 38(5 Suppl), S25–S33. 10.1016/j.ajic.2010.04.196 20569853

[jam15729-bib-0060] Xu, X. , Wang, Y. , Liao, S. , Wen, Z.T. & Fan, Y. (2012) Synthesis and characterization of antibacterial dental monomers and composites. Journal of Biomedical Materials Research, 100B(4), 1151–1162. 10.1002/jbm.b.32683 PMC3407682 22447582

[jam15729-bib-0061] Yu, L. , Li, K. , Zhang, J. , Jin, H. , Saleem, A. , Song, Q. et al. (2022) Antimicrobial peptides and macromolecules for combating microbial infections: from agents to interfaces. ACS Applied Bio Materials, 5(2), 366–393. 10.1021/acsabm.1c01132 35072444

[jam15729-bib-0062] Zapotoczna, M. , Murray, E.J. , Hogan, S. , Gara, J.P.O. , Chhabra, S.R. , Chan, W.C. et al. (2017) 5‐Hydroxyethyl‐3‐tetradecanoyltetramic acid represents a novel treatment for intravascular catheter infections due to *Staphylococcus aureus* . The Journal of Antimicrobial Chemotherapy, 72, 744–753. 10.1093/jac/dkw482 27999062 PMC5400099

[jam15729-bib-0063] Zavascki, A.P. , Goldani, L.Z. , Li, J. & Nation, R.L. (2007) Polymyxin B for the treatment of multidrug‐resistant pathogens: a critical review. The Journal of Antimicrobial Chemotherapy, 60(6), 1206–1215. 10.1093/jac/dkm357 17878146

[jam15729-bib-0064] Zhou, Q. , Fan, L. , Lai, X. , Tan, L. & Zhang, X. (2019) Estimating extra length of stay and risk factors of mortality attributable to healthcare‐associated infection at a Chinese university hospital: a multi‐state model. BMC Infectious Disease, 19, 975. 10.1186/s12879-019-4474-5 PMC6864951 31747887

